# Altered expression of topoisomerase II*α* contributes to cross-resistant to etoposide K562/MX2 cell line by aberrant methylation

**DOI:** 10.1038/sj.bjc.6602498

**Published:** 2005-03-29

**Authors:** T Asano, K Nakamura, H Fujii, N Horichi, T Ohmori, K Hasegawa, T Isoe, M Adachi, N Otake, Y Fukunaga

**Affiliations:** 1 Department of Pediatrics, Nippon Medical School, Tokyo, Japan; 2 Pharmaceutical Research Laboratory, Kirin Brewery Co., Ltd, Gunma, Japan; 3 The 1st Department of Internal Medicine, Showa University School of Medicine, Tokyo, Japan; 4 Department of Medical Oncology, Tochigi Cancer Center Hospital, Tochigi, Japan; 5 Pharmacology Division, National Cancer Center Institute, Tokyo, Japan; 6 School of Science and Engineering, Teikyo University, Tochigi, Japan

**Keywords:** K562, topoisomerase, MX2, etoposide, methylation

## Abstract

KRN 8602 (MX2) is a novel morpholino anthracycline derivative having the chemical structure 3′-deamino-3′-morpholino-13-deoxo-10-hydroxycarminomycin hydrochloride. To investigate the mechanisms of resistance to MX2, we established an MX2-resistant phenotype (K562/MX2) of the human myelogeneous leukaemia cell line (K562/P), by continuously exposing a suspension culture to increasing concentrations of MX2. K562/MX2 cells were more resistant to MX2 than the parent cells, and also showed cross-resistance to etoposide and doxorubicin. Topoisomerase (Topo) II*α* protein levels in K562/MX2 cells were lower of those in K562/P cells on immunoblot analysis and decreased expression of Topo II*α* mRNA was seen in K562/MX2 cells. Topoisomerase II catalytic activity was also reduced in the nuclear extracts from K562/MX2 cells when compared with K562/P cells. Aberrant methylated CpG of Topo II*α* gene was observed in K562/MX2 cells when compared with the parent line on methylation-specific restriction enzyme analysis. To overcome the drug resistance to MX2 and etoposide, we investigated treatment with 5-Aza-2′-deoxycytidine (5AZ), which is a demethylating agent, in K562/MX2 cells. 5-Aza-2′-deoxycytidine treatment increased Topo II*α* mRNA expression in K562/MX2 cells, but not in K562/P cells, and increased the cytotoxicity of MX2 and etoposide. Methylated CpG was decreased in K562/MX2 cells after 5AZ treatment. We concluded that the mechanism of drug resistance to MX2 and etoposide in K562/MX2 cells might be the combination of decreased expression of Topo II*α* gene and increased methylation, and that 5AZ could prove to be a novel treatment for etoposide-resistant cell lines, such as K562/MX2.

DNA topoisomerase II (Topo II) is an ATP-dependent enzyme that makes transient breaks in one segment of double-stranded DNA and places an intact duplex into the broken DNA before resealing the break, thus altering DNA topology (Osheroff, 1989; Wang, 1996). The activity of Topo II is important in DNA metabolism, as well as in forming the nuclear scaffold and nuclear matrix (Osheroff, 1989; Wang, 1996). The catalytic activity of Topo II in mammalian cells is mediated by two genetically distinct isoenzyme forms, designated Topo II*α* and Topo II*β*, (Mw: 170 000 and 180 000, respectively) (Wang, 1996). The two isoenzymes appear to differ in both the nuclear localisation and relative content during cell cycle transition and cell proliferation (Osheroff, 1989; Wang, 1996).

Topo II has also been identified as the cellular target for many clinically active antineoplastic agents, including amino acridines, anthracyclines and epipodophyllotoxins (Zwelling *et al*, 1989). These agents act by stabilizing the Topo II enzyme–DNA complex and the Topo II-associated DNA strand break, which is designated the cleavable complex (Liu, 1989). The cellular processing of these complexes results in the generation of DNA double-strand breaks that eventually leads to cell death (Zwelling *et al*, 1989). However, the frequent emergence of resistant cells during treatment with Topo II-active drugs is a serious problem in cancer therapy (Zwelling *et al*, 1989; Harris and Hochhauser, 1992; Zhang *et al*, 1999).

KRN 8602 (MX2) is a new morpholino anthracycline derivative with the chemical structure 3′-deamino-3′-morpholino-13-deoxo-10-hydroxycarminomycin hydrochloride, and has been shown to exhibit cytotoxic effects against tumour cells as a Topo II inhibitor (Watanabe *et al*, 1988). Owing to its highly lipophilic properties, MX2 rapidly diffuses through the cell membrane and reaches high intracellular concentration irrespective of P-glycoprotein expression levels (Watanabe *et al*, 1988). MX2 has thus been capable of antitumour effects superior to those of adriamycin (ADM) against several murine and human tumour cell lines, even against multidrug-resistance tumour cell lines that overexpress P-glycoprotein (Watanabe *et al*, 1991). As a result, MX2 is considered a candidate antitumour drug against multidrug resistant tumour cells.

In order to elucidate the cellular target of and resistance mechanisms against MX2, we established an MX2-resistant human leukaemia cell line (K562/MX2), which is eight-fold more resistant to MX2 than the parental K562 cell line (K562/P). We observed quantitative changes in Topo II*α* in K562/MX2 cells when compared with those in K562/P cells, whereas intracellular level of MX2 was higher than in K562/P cells. Increased methylated CpG islands in the Topo II*α* gene were observed in K562/MX2 cells on methylation-specific restriction enzyme analysis. To overcome drug resistance to MX2 and etoposide, we investigated treatment with 5-Aza-2′-deoxycytidine (5AZ), which is a demethylating agent, in K562/MX2 cells. 5-Aza-2′-deoxycytidine treatment increased Topo II*α* mRNA expression in K562/MX2 cells, but not in K562/P cells, and increased the cytotoxicity of MX2 and etoposide. This suggests that Topo II*α* might be the cellular target of MX2.

## MATERIALS AND METHODS

### Drugs and chemicals

MX2 was prepared as described previously (Watanabe *et al*, 1991), and its hydrochloride form was used in this study (Watanabe *et al*, 1988, 1991). Adriamycin, etoposide, VINCR istine (VCR) carboplatin and dimethyl sulphoxide (DMSO) were obtained from Wako Pure Chemical Industries, Ltd (Osaka, Japan). Phosphate-buffered saline without metal salt solution (PBS(−)) was from Nissui (Tokyo, Japan). RPMI 1640, Hanks' balanced salt solution without Ca^2+^ or Mg^2+^ (HBSS), foetal calf serum (FCS) and gentamicin were purchased from Life Technologies, Inc. (Gaithersbug, MD, USA).

### Cell lines

K562/P, parental cell line of human myelogeneous leukaemia and its ADM resistant subline K562/ADM were kindly provided by Dr T Tsuruo (Cancer Chemotherapy Center, Japanese Foundation for Cancer Research) and K562/P was obtained from American Type Culture Collection (Manassas VA, USA). K562/ADM exhibited a typical multidrug resistance phenotype. The growth medium for K562/P and K562/ADM was RPMI-1640 medium supplemented with 10% heat-inactivated foetal bovine serum and 100 *μ*g ml^−1^ of kanamycin or gentamicin (R10). Cells were maintained at 37°C in a humidified atmosphere containing 5% CO_2_. K562/ADM cells were maintained in an ADM concentration of 0.3 *μ*g ml^−1^. An MX2-resistant cell line was selected by stepwise and continuous exposure to MX2 using the limiting dilution method. Prior to use in each experiment, K562/MX2 was cultured without MX2 for 2 weeks. Cell sizes were determined using a Coulter channelizer C-256 system (Nikkaki, Tokyo). All cell lines were free from *mycoplasma* organisms, as confirmed by a MycoFluor™ Mycoplasma detection kit (Molecular Probes, Eugene, OR, USA).

### Cytotoxicity assay

Cytotoxicity was measured by trypan blue dye exclusion assay as described previously (Ishiwaka *et al*, 1994). Briefly, 1 × 10^6^ cells were incubated with various concentrations of anticancer drugs, including MX2, etoposide, ADM and VCR, for 72 h and viable cells were counted after trypan blue staining.

### Assays for cellular uptake and efflux of MX2 and ADM

Intracellular uptake and efflux of MX2 and ADM were determined by the method by Horichi *et al* (1990). To study uptake, each 1 × 10^6^ K562/P or K562/MX2 cells were incubated with 0.25 mmol of MX2 or ADM for 120 min. For efflux assay, tumour cells were further incubated in drug-free medium for 120 min. At each time point, 1 × 10^6^ cells were removed and washed twice with 10 ml of ice-cold PBS(−) and the incorporated MX2 or ADM was extracted with 100 *μ*l of DMSO, and cellular proteins were then precipitated with the addition of 0.4 ml of absolute methanol. The fluorescence intensity of the extracts was determined with a fluorescence spectrophotometer at excitation and emission wavelengths of 485 and 535 nm.

### P-glycoprotein expression in leukaemia cells by flow cytomery

Cells in various conditions were incubated with PE-labelled anti-human P-glycoprotein antibody (Immunotech, Marseille, France) for 30 min at room temperature, washed with PBS three times and analysed by flow cytometry (Coulter, CA, USA).

### Northern blot analysis and RT–PCR

RNA was extracted, electrophoresed, transferred and hybridised with a human Topo II*α* gene probe (generous gift of Dr L Liu, Robert Wood Johnson Medical School, University of Medicine and Dentistry of New Jersey, NJ, USA), Topo II*α* cDNA fragment, which was amplified from a single-stranded cDNA library using primer set 5′-GGAGAGCAGCAACAAAAACA-3′ (3953–3972) and 5′-CTTGCTTGTGACTGCTTTCG-3′ (4484–4503), *β*-actin probe (generous gift of Dr Eugenie S Kleinerman, University of Texas, MD Anderson Cancer Center), or human glyceraldehyde 3-phosphate dehydrogenase cDNA (Asano *et al*, 1996a). RT–PCR was performed according to the manufacturer's instructions (Takara, Ootsu). The primers used were 5′-CCGTGTACTCCAACGCTGC-3′ and 5′-CTGGACCGCTGACGCCGTGAC-3′ for multidrug resistance protein1 (MRP1), yielding a PCR product of 326 bp (Roller *et al*, 1999) and 5′-GTGGGGCGCCCCAGGCACA-3′ and 5′-CTCCTTAATGTCACGCACGATTTC-3′ for *β*-actin, yielding a PCR product 548 bp (Brenner *et al*, 1989).

### Immunoblot analysis of Topo II

K562/P, K562/ADM and K562/MX2 cells were pelleted (900 × **g**) and washed twice with cold PBS(−). Cells were resuspended at a density of 2 × 10^7^ cells ml^−1^ in 50 mM Tris HCl (pH 7.4), 2% SDS, 1% *β*-mercaptoethanol, 1 mM PMSF, 10 *μ*g ml^−1^ aprotinin and 10 *μ*g ml^−1^ leupeptin. Subsequently, cells were incubated at 65°C for 5 min and passed 15–20 times through a 25-gauge needle. Proteins from whole-cell lysate were prepared for SDS–polyacrylamide gel electrophoresis by addition of an equal volume of SDS sample buffer (4% SDS, 0.2 M DTT, 20% glycerol) and boiling for 5 min. Samples were electrophoresed (1 × 10^5^ cells lane^−1^) on 7% SDS–polyacrylamide gels and transferred onto nitrocellulose membranes as described previously (Zwelling *et al*, 1989; Harris and Hochhauser, 1992; Asano *et al*, 1996a; Zhang *et al*, 1999). Blots were probed with human Topo I antibody (TopoGEN, Inc., Columbus, OH, USA) and monoclonal antibodies 8D2, which recognise the *M*_r_ 170 000 form of the Topo II*α* enzyme (kindly provided by Dr A Kikuchi, Laboratory of Medical Micology, Research Institute of Disease Mechanism and Control, Nagoya University School of Medicine, Nagoya). Topo proteins were detected using the Amersham enhanced chemiluminescence detection system according to the protocols of the manufacturer. Autoradiograms were evaluated by densitometry in order to quantify the Topo signals.

### Topoisomerase decatenation assays

Crude nuclear extracts from K562/P, K562/ADM and K562/MX2 cells were prepared as described previously with some modification (Zwelling *et al*, 1989; Harris and Hochhauser, 1992; Zhang *et al*, 1999). Briefly, cells in the exponential phase of growth were washed three times with five volumes of PBS(−) containing 1 mM PMSF, 1 mM benzamidine and 1 *μ*g ml^−1^ soybean trypsin inhibitor, and were then centrifuged for 5 min at 1000 × *g*. Cells were resuspended in four volumes of 5 mM potassium phosphate (pH 7.0), 2 mM MgCl_2_, 0.1 mM EDTA, 1 mM PMSF, 1 mM benzamidine, 10 *μ*g ml^−1^ soybean trypsin inhibitor, 50 *μ*g ml^−1^ leupeptin and 10 mM 2-mercaptoethanol, and were stirred slowly at 4°C for 15 min. Nuclei were prepared by lysing cells with 10–15 strokes from a chilled Dounce homogenizer. The extent of cell lysis was monitored by microscopy. Cell lysate was centrifuged for 10 min at 1000 × **g**. Nuclei were washed twice with five volumes of 1 mM potassium phosphate (pH 6.5), 5 mM MgCl_2_, 1 mM EGTA, 10% glycerol, 100 mM NaCl, 1 mM PMSF, 1 mM benzamidine, 10 *μ*g ml^−1^ soybean trypsin inhibitor, 50 *μ*g ml^−1^ leupeptin and 10 mM 2-mercaptoethanol by centrifuging at 1000 × **g** for 5 min. Washed nuclei were resuspended in extraction buffer (5 mM potassium phosphate (pH 7.0), 2 mM MgCl_2_, 0.1 mM EDTA, 1 mM PMSF, 1 mM benzamidine, 10 *μ*g ml^−1^ soybean trypsin inhibitor, 50 *μ*g ml^−1^ leupeptin, 10 mM 2-mercaptoethanol and 10% glycerol). NaCl was added slowly to a final concentration of 0.35 M and topoisomerase was extracted for 60 min at 4°C with constant stirring. Extracts were centrifuged at 25 000 × **g** for 15 min, and the supernatants were used as topoisomerase extracts. Protein concentration in the final extracts was determined by the method of Bradford. Topoisomerase I activity was measured by the relaxation of supercoiled DNA using a Topoisomerase I Assay Kit from TopoGEN, Inc. (Columbus, OH, USA). Topoisomerase II catalytic activity was assayed by decatenation of kDNA into free mini circles using a Topoisomerase II Assay Kit from TopoGEN, Inc. Relaxation and decatenation were carried out using topoisomerase extracts, which contained 2–256 ng of total protein, from K562/P, K562/ADM and K562/MX2 with the appropriate supplements. Reaction products were analysed by agarose gel electrophoresis according to the manufacturer's instructions.

### Methylation status analysis using methylation-specific enzyme

In total, 1 *μg* of control, K562/P and K562/MX2 DNA was digested with 100 U of *Msp*I and *Hpa*II (Takara Shuzo, Ohtsu, Japan) at 37°C for 16 h. To analyse the methylation status of the Topo II*α* promoter region, restriction-digested DNA was analysed by PCR in a 25-*μ*l reaction mixture containing 1 *μ*mol l^−1^ of each sense and antisense primer, and 1 U of Taq DNA polymerase. PCR primers were as follows: 5′-AGGCAGATGCCAGAATCTGTT-3′, corresponding to −550 to −529, and 5′-AGGGCTCACTTGTTTTCTCGT-3′, corresponding to −284 to −263 (restriction site −489); and 5′-GCTCCCATTCCCCTCGCTAAC-3′, corresponding to −457 to −436, and 5′-AGGGCTCACTTGTTTTCTCGT-3′, corresponding to +26 to +47 (restriction site: −152), based on the sequence by Hochhauser *et al* (1992). PCR products were separated by electrophoresis on 2% agarose gels.

### 5-Aza-2′-deoxycytidine treatment

Approximately 1 × 10^8^ cells from various cell lines were grown on medium containing 2, or 10 *μ*M 5AZ for 3 days, and the medium and drug were replaced every 24 h.

## RESULTS

### Establishment of MX2 resistant cell line

K562/MX2 was developed by continuously exposing cells to gradually increasing doses of MX2. K562/MX2 is a subculture under continuous exposure to 0.1 *μ*M MX2. This resistant phenotype was also stable after more than 6 months of growth in drug-free medium. The doubling time of K562/MX2 (29 h) was slightly longer than that of the parent cell line K562/P (25 h) and that of the K562/ADM line (24 h). The diameter of K562/MX2 (8.7 *μ*m) was similar to that of parent cell line (8.6 *μ*m). K562/MX2 cells were resistant to MX2 and ADM and also showed cross-resistance to etoposide (Table 1).

### Accumulation and efflux of MX2 and ADM in K562/P and K562/MX2 cells

**Figure 1:**
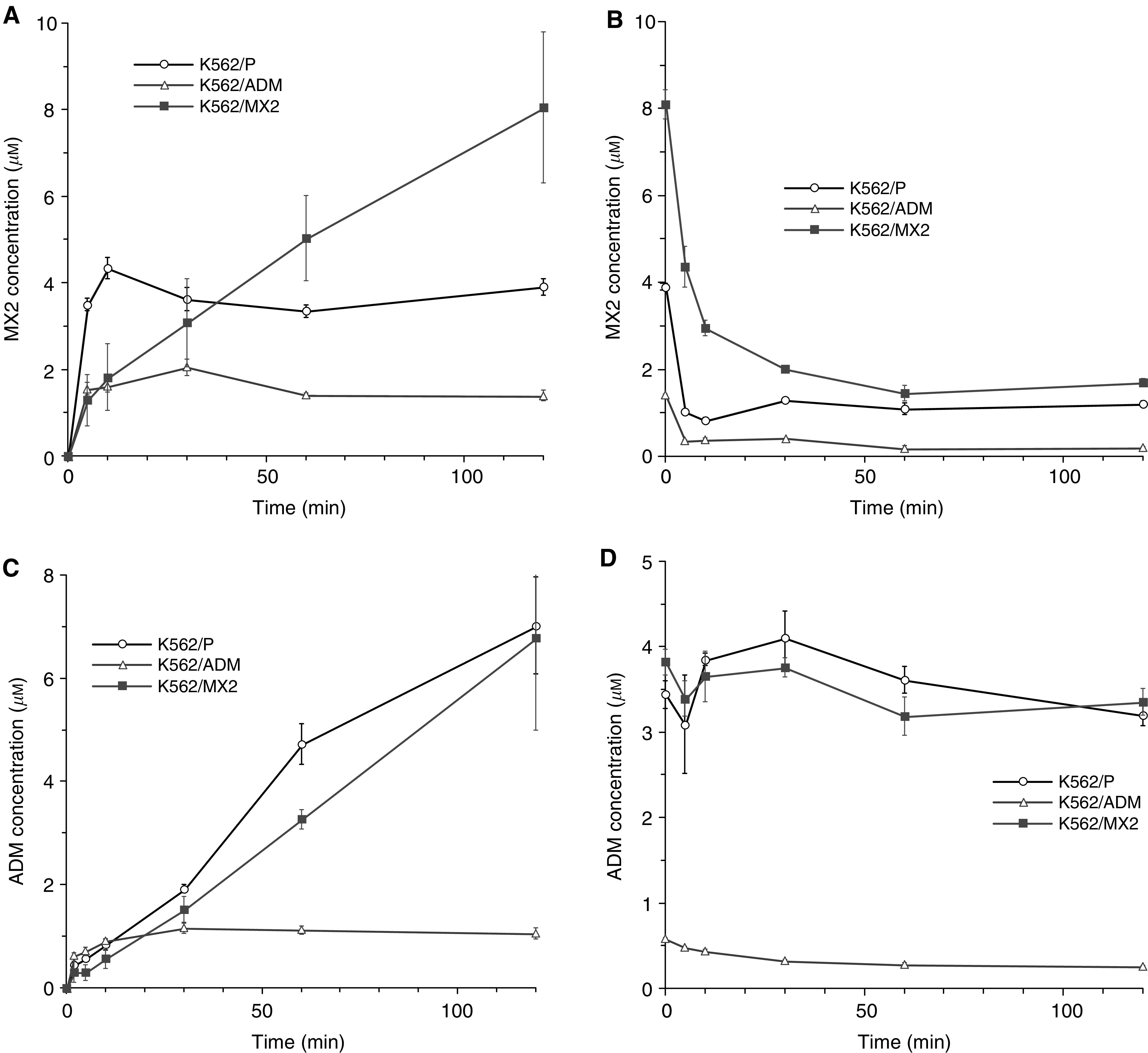
(**A**) Accumulation of MX2 in K562/P, K562/ADM and K562/MX cells. Intracellular accumulation of MX2 in K562/P, K562/ADM and K562/MX2 was measured according to Materials and Methods. Representative data from three independent experiments. Error bar showed s.d. (**B**) The efflux of MX2 in K562/P, K562/ADM and K562/MX2. The efflux of MX2 in K562/P, K562/ADM and K562/MX2 cells was measured according to Materials and Methods. Representative data from three independent experiments. Error bar showed s.d. (**C**) The accumulation of ADM in K562/P, K562/ADM and K562/MX cells. Intracellular accumulation of ADM in K562/P, K562/ADM and K562/MX2 was measured according to Materials and Methods. Representative data from three independent experiments. Error bar showed s.d. (**D**) The efflux of ADM in K562/P, K562/ADM and K562/MX2. The efflux of ADM in K562/P, K562/ADM and K562/MX2 cells was measured according to Materials and Methods. Representative data from three independent experiments. Error bar showed s.d.

In order to determine whether the resistance of K562/MX2 cells against MX2 and ADM was related to decreased intracellular drug concentration, the accumulation and efflux of the drugs in K562/MX2 were analysed. The uptake of MX2 in K562/P and K562/ADM cells reached a plateau within 30 min in the presence of 5 *μ*mol of drug over period 120 min, but K562/MX2 continuously showed increased MX2 concentration over the 120-min period (Figure 1A). The amount of MX2 quickly decreased in the K562/MX2 cell line after removal of MX2 from the culture media, and there was no difference in the concentration of MX2 between the three cell lines after the 120-min efflux period (Figure 1B). The intracellular concentration of ADM in K562/P and K562/MX2 continuously increased over the 120-min period. On the other hand, intracellular concentration of ADM in K562/ADM cells reached a plateau in 20 min (Figure 1C). Efflux of ADM was similar between K562/P and K562/MX2 cells, and was much slower than in K62/ADM cells (Figure 1D). P-glycoprotein expression was not detected in K562/P and K562/MX2 cells (data not shown). On the other hand, RNA expression of MRP1 was slightly higher in K562/MX2 and K562/ADM when compared with K562/P cells (data not shown). Incubation with 3 *μ*g ml^−1^ of indomethacin, an inhibitor of MRP1, for 72 h decreased MRP1 RNA expression in K562/MX2 and K562/ADM cells to a similar level as that in K562/P (data not shown). However, cytotoxicity of MX2 and etoposide in K562/MX2 cells treated with indomethacin was comparable to that in K562/MX2 cells without indomethacin treatment (Table 1). These results suggest that the resistance of K562/MX2 cells to etoposide and MX2 is not associated with P-glycoprotein, and that MRP1 contributes little to MX2 and etoposide resistance.

### Topo II*α* expression of in K562/P, K562/MX2 and K562/ADM cells

**Figure 2:**
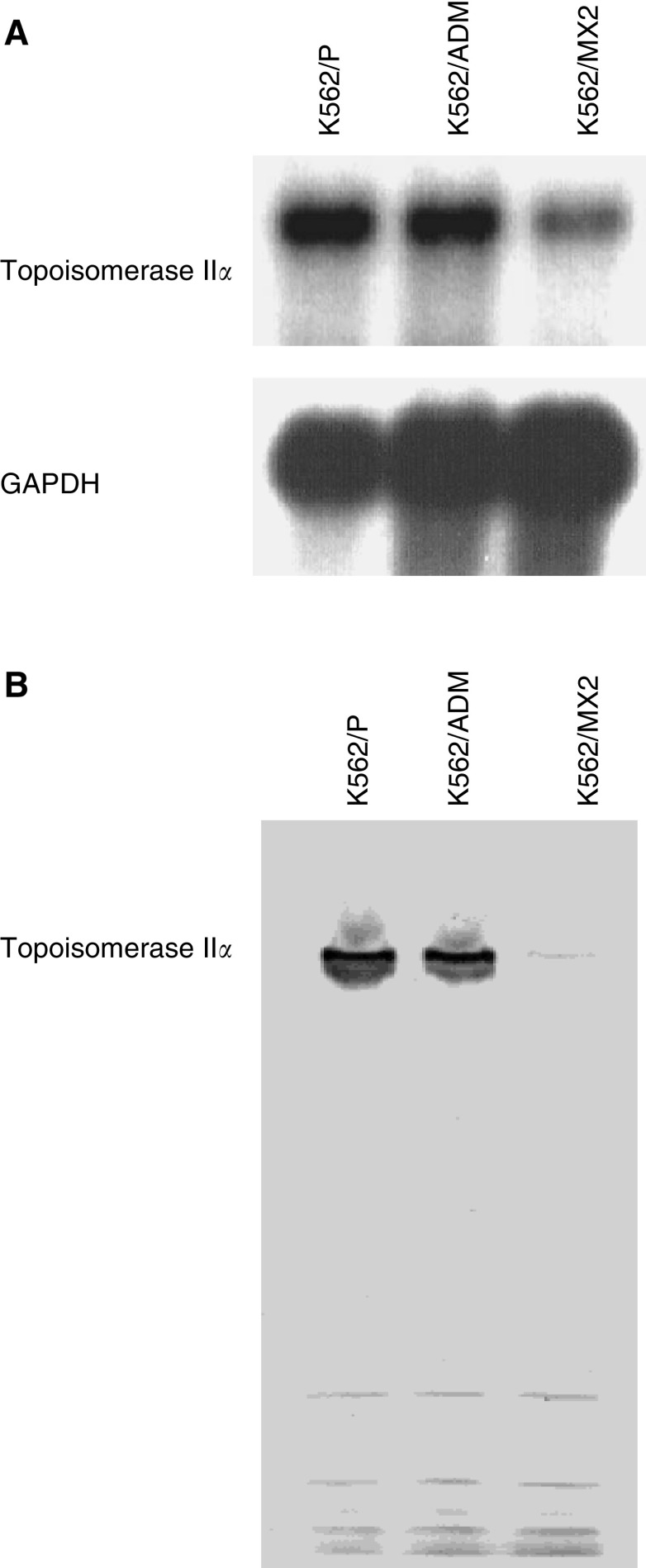
(**A**) Expression of topoisomerase II*α* mRNA in K562/P, K562/MX2 and K562/ADM. Human topoisomerase II*α* mRNA expression in K562/P, K562/ADM and K562/MX2 was measured. Significantly decreased expression of human topoisomerase II*α* was observed in K562/MX2 cells. In K562/ADM cells, slightly decreased expression of human topoisomerase II*α* was observed. Representative data from three independent experiments. (**B**) Expression of topoisomerase II*α* protein in K562/P, K562/MX2 and K562/ADM: Decreased expression of human topoisomerase II*α* protein in K562/MX2 was observed. Representative data from three independent experiments.

Topo II*α* mRNA expression was significantly lower in K562/MX2 cells and slightly lower in K562/ADM cells when compared with K562/P cells (Figure 2A).

### Topo I and Topo II protein in K562/P, K562/MX2 and K562/ADM cells

Immunoblot analysis of whole-cell lysates from K562/P, K562/MX2 and K562/ADM cells revealed identical Topo I expression levels in all cell lines (data not shown). Immunoblot analysis of whole-cell lysates from K562/P, K562/MX2 and K562/ADM cells confirmed the specific 170-kDa band of Topo II*α* in all cell lines, but K562/MX2 and K562/ADM cells showed lower levels than K562/P cells (Figure 2B).

### Topoisomerase decatenating activity in K562/P, K562/MX2 and K562/ADM cells

**Figure 3:**
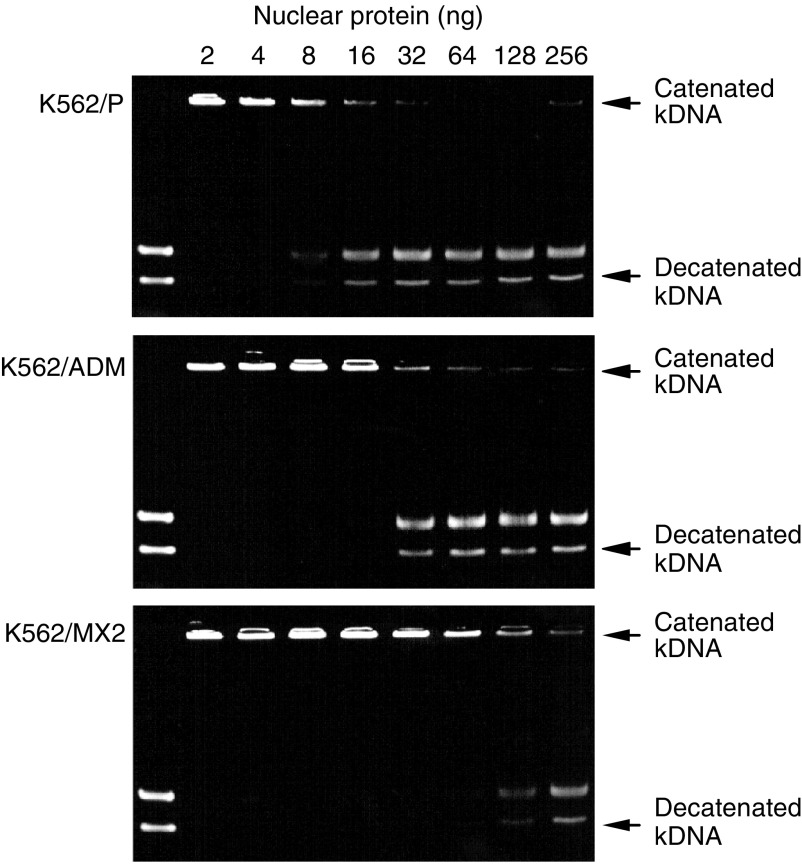
Topo II-mediated decantenation activity in nuclear extracts from in K562/P, K562/MX2 and K562/ADM cells. Decreased decantenation activity in K562/MX2was observed compared to in K562/P and K562/ADM cells. Representative data from three independent experiments.

In several mammalian cell lines selected for resistance to Topo II inhibitors, alterations in cellular Topo II protein activity, content or affinity for drugs have been demonstrated (Zwelling *et al*, 1989; Asano *et al*, 1996b, 1996c; Scheltema *et al*, 1997; Zhang *et al*, 1999). MX2 inhibits Topo II decatenating activity *in vitro* (Horichi *et al*, 1990). We thus investigated Topo I and Topo II catalytic activities in nuclear extracts from K562/P, K562/MX2 and K562/ADR cells. There were no differences among those cells in Topo I-mediated relaxation activity in nuclear extracts from exponentially growing cells (data not shown). On the other hand, Topo II activity in nuclear proteins from K562/MX2 cells was about 16-fold lower than that in comparable K562/P extracts (Figure 3). In K562/ADM nuclear extracts, the catalytic activity of Topo II was only two-fold lower than that in K562/P extracts (Figure 3).

### Increased methylation of CpG in Topo II*α* gene in K562/MX2 cells based on methylation-specific enzyme analysis

Increased methylation of the CpG site was confirmed in K562/MX2 cells at the proximal promoter region (position: −152, Figure 4), but not at the distal promoter region (position: −489, data not shown) of the Topo II*α* gene by methylation-specific enzyme analysis. There were no mutations in the promoter region (position −566 to +22) of the human Topo II*α* gene in K562/P and K562/MX2 cells, as compared to the published sequence (Hochhauser *et al*, 1992).

### Topo II*α* mRNA expression after 5AZ treatment in K562/P and K562/MX2 cells

We then determined whether aberrant methylation might confer decreased expression of the Topo II*α* gene. 5-Aza-2′-deoxycytidine treatment was performed in K562/P and K562/MX2 cells. Increased human Topo II*α* mRNA expression was observed after treatment with 2 or 10 *μ*M of 5AZ in K562/MX2 cells, but not in K562/P cells (Figure 5). However, the intensity of Topo II*α* mRNA in K562/MX2 cells remained lower than that in K562/P cells.

### Decreased CpG methylation in Topo II*α* in K562/MX2 cells treated with 5AZ

**Figure 4:**
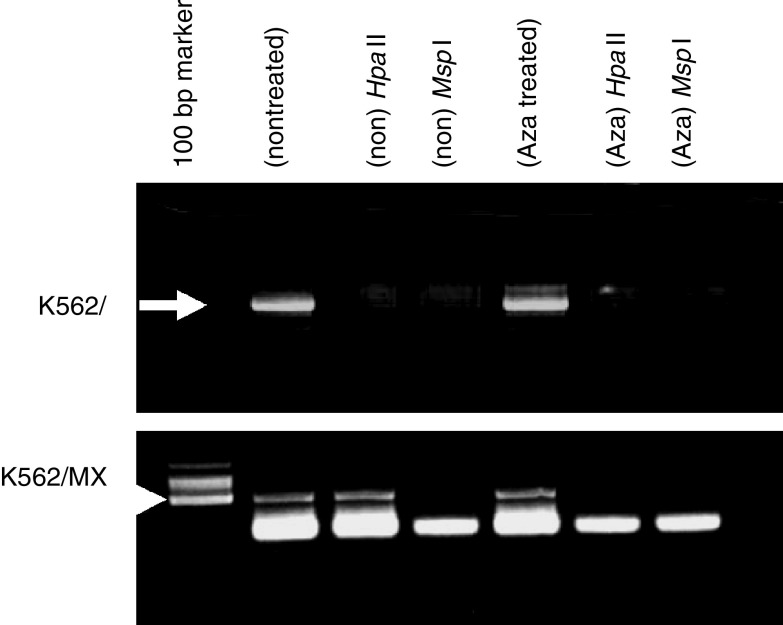
Methylation status of promoter region in K562/P and K562/MX2 cells by methylation-specific restriction enzyme analysis with or without 5-Aza-2′-deoxycytidine treatment. Aberrant methylation of CpG site was observed in K562/MX2 cells at the proximal promoter region (position: −152) by methylation-specific enzyme analysis (arrow head). PCR products in K562/P cells showed arrow. Representative data from five independent experiments.

**Figure 5:**
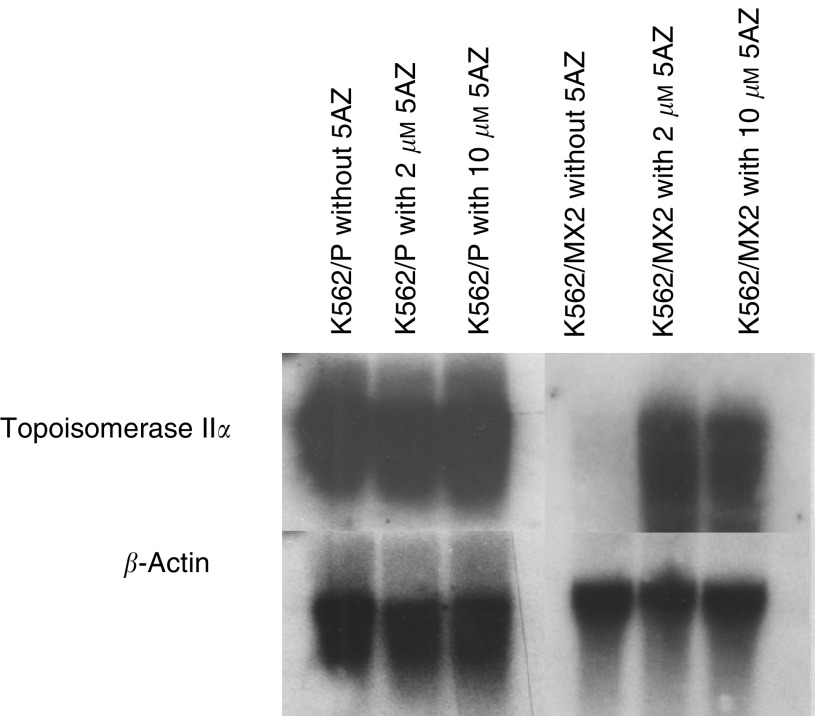
Topoisomerase II*α* mRNA expression with 5-Aza-2′-deoxycytidine treatment in K562/P and K562/MX2 cells. Increased expression of topoisomerase II*α* RNA in K562/MX2 treated with 5-Aza-2′-deoxycytidine was observed. However, increased expression in 5AZ treated K562/MX2 cells was not fully restored compared inK562/P cells. Representative data from six independent experiments.

In K562/MX2 cells treated with 5AZ, fewer methylated CpG sites were observed on methylation-specific enzyme analysis (Figure 4).

### Effect of 5AZ treatment on MX2 and etoposide cytotoxicity in K562/P, K562/ADM and K562-MX2 cells

Increased cytotoxicity of MX2 and etoposide was observed in 5AZ-treated K562/MX2 cells, but was not observed in 5AZ-treated K562/P and K562/ADM cells (Table 1). Also carboplatin, which act as an independent mechanism of Topo II, showed no enhanced sensitivity with 5AZ treatment. In 5AZ-treated K562/ADM cells, decreased resistance against ADM and VCR were observed as same as previous literature (Efferth *et al*, 2001). Under 10 *μ*M of 5AZ treatment for 1 week showed no cytotoxic effect against K562/P, K562/ADM and K562/MX2 cells (data not shown).

## DISCUSSION

Drug resistance is a major obstacle in cancer therapy. The molecular target of drug action for amino acridines, anthracyclines and epipodophyllotoxins has been reported to be topoisomerase (Zwelling *et al*, 1989). Multiple resistance to Topo II poisons exist in two major forms: one is attributable to an efflux pump in the cell membrane that lowers the steady-state concentration of the drug at the target site, and in the other form, the activity and sensitivity of the target enzyme Topo II itself are decreased by downregulation or mutation (Kellner *et al*, 2002).

KRN 8602 (MX2) is a novel morpholino anthracycline derivative with the chemical structure 3′-deamino-3′-morpholino-13-deoxo-10-hydroxycarminomycin hydrochloride, and has been demonstrated to have cytotoxic effects against tumour cells as a Topo II inhibitor (Watanabe *et al*, 1988). MX2 is highly lipophilic and has been shown to rapidly diffuse through the cell membrane and reach high intracellular concentrations regardless of P-glycoprotein expression levels (Watanabe *et al*, 1988). To elucidate the cellular target of and resistance mechanisms against MX2, we established an MX2-resistant human leukaemia cell line (K562/MX2) that is more resistant to MX2 and etoposide than the parental K562 cell line (K562/P), and we investigated the mechanisms Topo II-targeting drugs in K562/MX2 cells.

We found that the accumulation and efflux of MX2 were marginally different in the MX2-resistant cell line. P-glycoprotein expression was similar in both K562/P and K562/MX2 cells. The role of the MRP1 gene is possibly marginal because indomethacin, an inhibitor of the MRP1 gene, did not alter the sensitivity to MX2 and etoposide in K562/MX2 cells. The resistance of numerous cell lines and clinical samples to doxorubicin has been found to be MDR1 or MRP1 mediated (Legrand *et al*, 1998, 1999). Based on the present results, it is reasonable to conclude that the resistance of K562/MX2 to etoposide, and MX2 does not involve MDR1 or MRP1.

On the other hand, we observed decreased expression of the Topo II*α* gene in K562/MX2 cells, but found no change in Topo I gene expression. From a clinical perspective, mutations in Topo II do not seem to have a major role in resistance (Kudo *et al*, 1996; Kellner *et al*, 2002). In addition to these findings, forced induction of Topo II*α* gene expression in etoposide-resistant cell lines using a dexamethasone inducible vector or a recombinant adenovirus vector containing normal human or drosophila Topo II*α* overcame etoposide resistance (Asano *et al*, 1996a, 1996b and 1996c; Zhang *et al*, 1999). Decreased expression of human Topo II*α* might therefore be a major factor in drug resistance to etoposide and other drugs. When analyzing the mechanisms of decreased Topo II*α* expression, increased methylation of CpG islands in the Topo II*α* gene were observed in K562/MX2 cells by methylation-specific restriction enzyme analysis. Aberrant methylation of several genes has been frequently reported in cancer development (Roman-Gomez *et al*, 2002). With regard to drug resistance, the several genes in which methylation was found to be related to drug resistance, such as MDR1 (Nakayama *et al*, 1998), caspase-8 (Worm and Guldberg, 2002; Paz *et al*, 2003), *O*(6)-methylguanine-DNA methyltransferase gene (Christmann *et al*, 2001), glutathione-*S* transferase P1 (Esteller *et al*, 1998) and hMLH1 gene (Plumb *et al*, 2000). The present results might be suggestive of a novel methylation-related mechanism of drug resistance. Interestingly, this resistance seems to have occurred via increased methylation of the target gene as well as decreased expression of a resistance-related gene, which is in complete contrast to resistance mechanisms involving the MDR1 gene.

In order to overcome resistance to MX2, we investigated treatment with 5AZ, which is a demethylating agent, in K562/MX2 cells. 5-Aza-2′-deoxycytidine treatment increased Topo II*α* mRNA expression in K562/MX2 cells, but not in K562/P cells, and increased cytotoxicity to MX2 and etoposide. CpG methylation was decreased in K562/MX2 cells after 5AZ treatment. Furthermore, 5AZ treatment increased mRNA expression of Topo II*α* and cytotoxicity to MX2 and etoposide in K562/MX cells. This suggests that Topo II*α* might be the cellular target of MX2. These findings were also observed in an etoposide-resistant breast cancer cell line (Asano, unpublished results). 5-Aza-2′-deoxycytidine also increased expression of many other genes in cancer cells, such as caspase-8 (Worm and Guldberg, 2002; Paz *et al*, 2003) and hMLH gene (Plumb *et al*, 2000). Such altered expression of genes by 5AZ treatment might contribute to restore the drug resistance in resistant cell lines. 5-Aza-2′-deoxycytidine is now used to treat myelodysplastic syndrome in clinical settings (Kornblith *et al*, 2002; Silverman *et al*, 2002). Based on the present results, we believe that 5AZ also might be applicable to patients exhibiting drug resistance, particularly to MX2 and etoposide.

In conclusion, the present MX2-resistant phenotype exhibits decreased expression of the topoisomerase II*α* gene together with increased methylation of this gene.

## Figures and Tables

**Table 1:** IC_50_ against MX2 and etoposide, adriamycin, and vincristine with or without indomethacin, 5-Aza-2′-deoxycytidine (5AZ) treatment

	**MX2 (nM)**	**Etoposide (nM)**	**Adriamycin (nM)**	**Vincristine (nM)**	**Carboplatin (*μ*M)**
K562/P	30±4	10±4	20±3	2.0±2.1	20±5.1
K562/P with indomethacin	28±7	9±5	18±3	1.8±2.0	ND
K562/P with 2 *μ*M 5AZ	28±5	8±5	ND	ND	ND
K562/P with 10 *μ*M 5AZ	29±6	7±4	15.0±11.0	1.8±2.9	22±4.8
K562/MX2	200±23*	94±15*	150±20*	2.3±1.8	18±6.0
K562/MX2 with indomethacin	190±50*	90±25*	100±25*	1.8±1.0	ND
K562/MX2 with 2 *μ*M 5AZ	50±12^a^	10±5^a^	ND	ND	ND
K562/MX2 with 10 *μ*M 5AZ	46±9^a^	8±5^a^	130±10.7	2.0±1.0	20.5±4.8
K562/ADM	28±9.3	8.2±3.0	250±50*	20.2±8.1*	21±6.8
K562/ADM with indomethacin	ND	ND	50±50**	5±3**	ND
K562/ADM with 10 *μ*M 5AZ	30±8.8	9.3±2.5	100±20.8^a^	5.0±2.8^a^	20±5.0

ND=not done. IC_50_ was calculated from cytotoxicity against various drugs. Data were average±s.d. from five independent experiments.

* *P*<0.05, cytotoxicity in K562/P *vs* K562/MX2, or K562/ADM cells.

** *P*<0.05, cytotoxicity in K562 cells with *vs* without indomethacin treatment.

a Cytotoxicity in K562 cells with *vs* without 5AZ treatment.
